# Effects of DNMT1 silencing on malignant phenotype and methylated gene expression in cervical cancer cells

**DOI:** 10.1186/1756-9966-30-98

**Published:** 2011-10-17

**Authors:** Yi Zhang, Fu-qiang Chen, Ye-hong Sun, Shu-yan Zhou, Ti-yuan Li, Rui Chen

**Affiliations:** 1 The Second Medical College, Jinan University, Shenzhen Clinical Medical Research Center, Shenzhen People's Hospital, 518020, Shenzhen, PR China; 2 The Pharmacy College, Jinan University, 510632, Guangzhou, PR China

## Abstract

**Background:**

DNA methylation has been widely used in classification, early diagnosis, therapy and prediction of metastasis as well as recurrence of cervical cancer. DNMT methyltransferase 1 (DNMT1), which plays a significant role in maintaining DNA methylation status and regulating the expression of tumor suppressor genes. The aim of this research was to investigate the relationship between DNMT1 and abnormal methylation of tumor suppressor genes and malignant phenotype in cervical cancer.

**Methods:**

Levels of DNMT1 mRNA and protein were detected using qPCR and Western blot, respectively. Cell proliferation was analyzed by MTT and apoptosis was performed by Annexin V-FITC/PI double staining flow cytometry, respectively. MeDIP-qPCR and qPCR were performed to measure demethylation status and mRNA re-expression level of 7 tumor-suppressor genes (CCNA1, CHFR, FHIT, PAX1, PTEN, SFRP4, TSLC1) in Hela and Siha cells after silencing DNMT1.

**Results:**

The average expression levels of DNMT1 mRNA and protein in Hela and Siha cells were decreased significantly compared with control group. The flow cytometry and MTT results showed that Hela and Siha cells apoptosis rates and cell viabilities were 19.4 ± 2.90%, 25.7 ± 3.92% as well as 86.7 ± 3.12%, 84.16 ± 2.67% respectively 48 h after transfection (*P *< 0.01). Furthermore, the promoter methylation of five tumor suppressor genes was decreased with the increased mRNA expression after silencing DNMT1, whereas there were no significant changes in PTEN and FHIT genes in Hela cells, and CHFR and FHIT genes in Siha cells.

**Conclusions:**

Our experimental results demonstrate that methylation status of DNMT1 can influence several important tumor suppressor genes activity in cervical tumorigenesis and may have the potential to become an effective target for treatment of cervical cancer.

## Background

Cervical cancer is the second most common cancer in women worldwide and the leading cause of cancer deaths in women in developing countries. It is obviously that many genetic and epigenetic alternations occur during cervical tumorigenesis. Among those changes, aberrant promoter methylation of tumor-suppressor genes gives rise to its silencing functions and results in the significant carcinogenesis of cervical cancer.

Currently, the known repressor genes are related to cervical cancer including CCNA1, CHFR, FHIT, PAX1, PTEN, SFRP4, TSLC1 and etc [1]. All these genes mentioned above have performed a wide variety of functions to regulate the transcription and expression, any of which down-regulation as well as promoter hypermethylation will lead to the precursor lesions in cervical development and malignant transformation. DNA methylation is catalyzed by several DNA methyltransferases, including DNMT1, DNMT3a, DNMT3b and etc. DNMT1 is responsible for precise duplicating and maintaining the pre-existing DNA methylation patterns after replication. As reported by Szyf [2], DNMT1 inhibited the transcription of tumor suppressor genes and facilitated the formation of tumorigenesis, which linked to the development of cervical cancer. Meanwhile, Inhibition of DNMT1 activity could reduce hypermethylation of repressive genes and promote its re-expression, and reverse phenotype of malignant tumor. Thus, specific inhibition of DNMT1 could be one strategy for cervical therapy.

In our study, we detected the demethylation and re-expression levels of seven cervical cancer suppressor genes with DNMT1 silencing in Hela and Siha cells. The aim was to elucidate the relations between DNMT1 and abnormal methylation of these genes' promoter as well as the malignant phenotype of tumor cells, which might contribute to the investigations of functions and regulation roles of DNMT1 in cervical cancer.

## Materials and methods

### Cell culture and transfection

The Hela and Siha human cervical cancer cells lines were obtained from American Type Culture Collection (Manassas, VA, USA). Lipofectamine TM2000 was purchased from Invitrogen Co. These cells grown in Dulbeco's Modified Eagle Medium (DMEM) supplemented with 10% fetal bovine serum and incubated at 37°C in a humidified chamber with 5% CO_2_. The siRNA primer sequences for DNMT1 were 5'-UUAUGUUGCUCACAAACUUCUUGUC-3' (forward) and 5'-GACAAGAAG UUUGUGAGCAACAUAA-3' (reverse), which were custom synthesized by Shanghai Sangon (Shanghai, China). After transfection, the inhibition efficiency was examined using quantitative polymerase chain reaction (qPCR). Transfections were performed with Lipfectamine TM2000 according to the protocol (Invitrogen Co.).

### Real-time qPCR assay

QPCR was used to analyze mRNA expression level of DNMT1. Total RNA was extracted using Trizol reagent and reversely transcribed into cDNA. The primers for DNMT1 were 5'-AACCTTCACCTAGCCCCAG-3' (forward) and 5'-CTCATCCGATTTGGCTCTTCA-3'(reverse); for GAPDH were 5'-CAGCCTCAAGATCATCAGCA-3'(forward) and 5'-TGTGGTCATGAGTCCTTCCA-3' (reverse). QPCR was performed in a 20 μl volume containing 1 μl cDNA template, 10 μl SYBR Green Real-time PCR Master Mix and 1 μl of each primer. Levels of seven tumor suppressor genes mRNA expression were also assayed with qPCR. This cycle was defined at 95°C for 5 min, followed by 35 cycles of denaturing at 95°C for 45s, annealing at 59°C for 35 s and extension at 72°C for 1 min, and followed by the final extension at 72°C for 10 min. The primers were shown in Table 1 and Table 2.

**Table 1:** Primers used in RNA expression

gene		Sequences	Tm (°C)	Product Size(bp)
QPCR	GAPDH	F:5'GGGAAACTGTGGCGTGAT3' R:5'GAGTGGGTGTCGCTGTTGA3'	59	299
	FHIT	F:5'GGAGATCAGAGGAGGAAATGG3' R:5'GGGAGTTGGAGTGACCGAG3'	59	233
	PTEN	F:5'ACACGACGGGAAGACAAGTT3' R:5'CTGGTCCTGGTATGAAGAATG3'	59	157
	CHFR	F:5'GCGTAGAAATGCCCAAACC3' R:5'TCCATCCAGCCCGAGTAGC3'	59	171
	SFRP4	F:5'GGCCTCTTGATGTTGACTGTAA3' R:5'GAGGGATGGGTGATGAGGA3'	59	204
	PAX1	F:5'GGTAGGAGTAGGGAGCACAGG3' R:5'CAAGTGTTGCGAGTGGAGG3'	59	100
	TSLC1	F:5'TTATTTCAGGGACTTCAGGC3' R:5'TTCCACCGCAGTGTCTTTC3'	59	223
	CCNA1	F:5'GCCTGGCAAACTATACTGTGAAC3' R:5'GTGCAGAAGCCTATGACGATTA3'	59	295

**Table 2:** Primers used in MeDIP-qPCR assay

gene		Sequences	Tm (°C)	Product Size(bp)
MSP	FHIT	F:5'GAAAGCCATAGTGACAGTAACCC3' R:5'AAAGCCAAAGATTGTGCGATT3'	59	121
	CCNA1	F:5'CTCCCGAGCCAGGGTTCT3' R:5'CGTTCTCCCAACAGCCGC3'	59	76
	PTEN	F:5'GAGCGAATGCAGTCCACG3' R:5'AGGCAGGGTAGGCTGTTGT3'	59	232
	CHFR	F:5'TTGCCTCAGTATCTCACTTCTT3' R:5'TCGCCGTCTTTACTCCTCT3'	59	118
	SFRP4	F:5'CCCCATTCTTTCCCACCTC3' R:5'TCGCCTGAAGCCATCGTC3'	59	164
	PAX1	F:5'AGGAGACCCTGGCATCTTTG3' R:5'GACGGCGGCTGCTTACTT3'	59	168
	TSLC1	F:5'GGGAGAACGGCGAGTTTAG3' R:5'GGCTGAGGGCATCTGTGAG3'	59	215

### Western blot analysis

Cells were harvested and rinsed twice in ice-cold PBS, and kept on ice for 30 min in cell lysis buffer containing 1 mM PMSF while agitating constantly, and insoluble cell debris was discarded by centrifugation for 10 min at 12,000 rpm at 4°C. The protein samples were separated with 12% SDS-PAGE and subsequently transferred to PVDF membranes (Millipore). Membranes were blocked with 5% nonfat dry milk solution either at room temperature for 2 h, and incubated with Rabbit anti-DNMT1 and secondary antibody at 37°C for 2 h respectively. The Membranes were stained with an enhanced chemiluminescence solution. Band intensities are normalized to β-actin as a loading control.

### Annexin V-FITC/PI staining and flow cytometry

Cell cycle analysis: Cells were digested by typsin (0.25%) and fixed with cold 70% ethanol at 48 h after transfection. After washed in phosphate-buffered saline, samples were incubated with 100 μl RNase A at 37°C for 30 min and stained with 400 μl propidium iodide (Sigma). Flow cytometric analysis was performed at 488 nm to determine the DNA contents.

Apoptosis analysis: Cells were harvested as described above. After adding of 10 μl Binding reagent and 1.25 μl Annexin V-FITC, samples were suspended in 0.5 ml cold 1 × Binding Buffer and stained with 10 μl PI. The samples were then analyzed for apoptosis by flow cytometry.

### MTT assay

Cellular proliferation was measured using MTT assay. 10^4 ^cells were seeded in 96-well plates and cultured with siRNA-DNMT1 at 37°C in a humid chamber with 5% CO_2 _for 24 h. 50 μl 1 × MTT was then added to each well and incubated with cells at 37°C for 4 h. After removal of supernatant, 150 μl DMSO were added to each well. The optical density (OD) was measured at 550 nm. The percentage of viability was calculated according to the following formula: viability% = T/C×100%, where T and C refer to the absorbance of transfection group and cell control, respectively.

### MeDIP-qPCR assay

Transfections were performed as described above. MeDIP assay combined with qPCR were used to quantitatively assess the status of demethylation. Hela and Siha cells were transfected with siRNA and treated with 1.0 μM 5-az-dC (Sigma) respectively, and harvested at 72 h after incubation. Genomic DNA was extracted and randomly sheared to an average length of 0.2-1.0 kb by sonication. Dilution buffer and 60 μl Protein G Magnetic Bead suspension were added into the fragmented DNA and allowed for more than 10 min of incubation. DNA was then incubated overnight at 4°C with 8 μg antibody (Epigentek) against 5-methylcytosine, followed by 2 h incubation with Mouse-IgG magnetic beads at 4°C. The methylated DNA/antibody complexes were then washed with 1 ml cold WB1, WB2 and WB3 buffer. Purified DNA was analyzed by qPCR on an Applied Biosystems 7500 Real-Time PCR System. Real-time PCR was performed in a total 8 μl volume containing 1 μl of DNA template, 5 μl of 2 × Master Mix, 1 μl ddH_2_O and 1 μl of each primer. The relative changes in the extent of promoter methylation were determined by measuring the amount of promoter in immunoprecipitated DNA after normalization to the input DNA: %(MeDNA-IP/Input) = 2^[(Ct(input)-Ct(MeDNA-IP)×100.

### Statistic analysis

Statistical analyses were performed with SPSS version 13.0(SPSS, Chicago, USA). Quantitative results were given as mean ± SD and statistical analysis was carried out by t-test. *P *values less than 0.05 were considered as statistically significant.

## Results

### Effects of siRNA on DNMT1 mRNA and protein level

**Figure 1:**
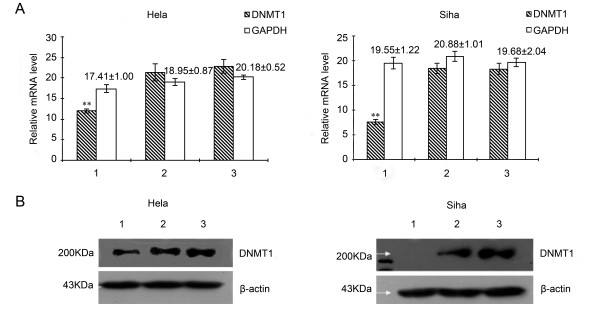
**Effects of siRNA on DNMT1 mRNA and protein expression**. (A): mRNA expression levels of DNMT1 in Hela and Siha cells were examined by qPCR. Compared with control group, Hela and Siha cells transfected with DNMT1-siRNA displayed lower level of mRNA expression (**P < 0.01). (B): DNMT1 protein levels in Hela and Siha cells were determined by western blot. The protein level of DNMT1 decreased significantly compared with control group and blank control. (1: transfection group (DNMT1-siRNA); 2: control group (negative siRNA); 3: blank group (Lipo2000), n = 3).

QPCR and western blot were performed to analyze the mRNA and protein expression levels of DNMT1 in Hela and Siha cells at 72 h after transfection. As shown in Figure 1A, Hela and Siha cells transfected with DNMT1-siRNA (transfection group) displayed lower level of mRNA expression (*P *< 0.01), with inhibitory ratios of 56.21% and 41.31% respectively compared with control group (negative siRNA). No significant change in DNMT1 mRNA expression was found between control group and blank control (Lipo 2000). The transcript quantity of GAPDH in transfection group, control group and blank control did not change significantly. Figure 1B showed the DNMT1 protein expression levels in Hela and Siha cells at 72 h after transfected with DNMT1-siRNA. The protein level of DNMT1 decreased significantly compared with control group and blank control (*P *< 0.01). The inhibitory ratios of DNMT1 protein level in Hela and Siha cells were 50.31% and 99.76%, respectively.

### Effects of DNMT1 silencing on cell cycle and apoptosis

**Figure 2:**
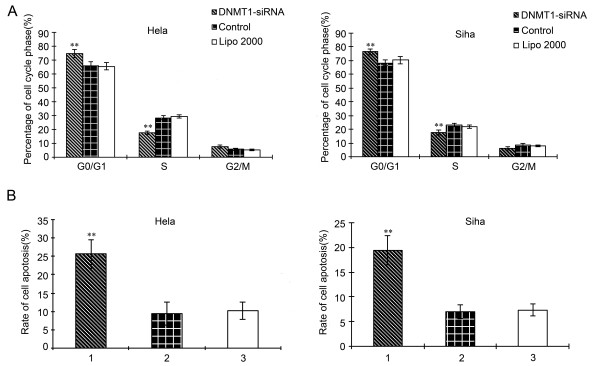
**Effects of DNMT1 silencing on cell cycle and apoptosis**. (A): Phases of cell cycle of Hela and Siha cells were analyzed by flow cytometry assay at 48 h after transfection (**P < 0.01). (B): Apoptosis of Hela and Siha cells was analyzed by flow cytometry assay at 48 h after transfection (**P < 0.01). (1: transfection group (DNMT1-siRNA); 2: control group (negative siRNA); 3: blank group (Lipo2000), n = 3).

The G0/G1 ratio (74.72 ± 3.17%) of Hela cells in transfection group was higher than that in control group (65.88 ± 3.23%) (*P *< 0.01), and cells at S phase were fewer compared with control group. Meanwhile, The G0/G1 ratio (76.43 ± 2.20%) of Siha cells in transfection group displayed significantly higher compared with control group (66.4 ± 1.99%) (*P *< 0.01), while cells at S phase were fewer than those in control group. No significant changes in G0/G1 ratio or cells at S phase were detected between the control group and blank control (Figure 2A). Furthermore, as shown in Figure 2B, the apoptosis of Hela cells in transfection group was significantly higher than that in control group (*P *< 0.01). Similar results were observed in Siha cells.

### Effects of DNMT1 silencing on cell growth and proliferation

**Figure 3:**
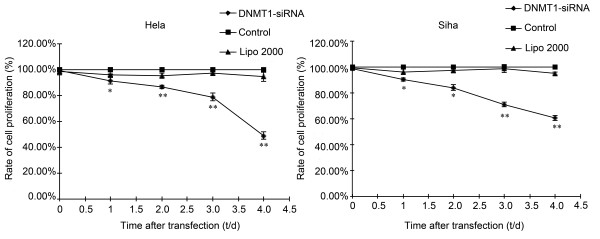
**Viability of Hela and Siha cells at different time after transfection determined by MTT assay**. Viabilities of Hela and Siha cells in transfection group were 91.47%, 86.74%, 78.92%, 48.98% and 90.45%, 84.16%, 71.09%, 60.47% at 24, 48, 72 and 96 h, respectively. (n = 3, *P < 0.05, **P < 0.01, compared with control group).

Cell growth and proliferation of Hela and Siha cells were examined using MTT assay. As shown in Figure 3, viabilities of Hela cells in transfection group were 91.47%, 86.74%, 78.92% and 48.98% at 24, 48, 72 and 96 h, respectively (*P *< 0.05) compared with control group at each time point. We observed the similar results in Siha cells with viabilities of 90.45%, 84.16%, 71.09% and 60.47% at 24, 48, 72 and 96 h after transfection, respectively (*P *< 0.05) compared with control group at each time point.

### Effects of DNMT1 silencing on gene demethylation and mRNA expression level in Hela cell

**Figure 4:**
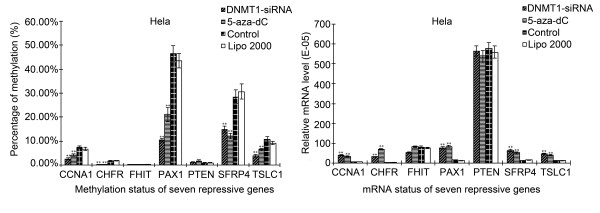
**Effects of DNMT1 silencing on gene methylation and mRNA expression of seven tumor suppressor genes in Hela cells assayed by MeDIP combined with Real-Time PCR**. Except for FHIT and PTEN, the rest five suppressor genes CCNA1, CHFR, PAX1, SFRP4 and TSLC1 in transfected group displayed lower level of methylation with increased mRNA expression when compared with control group. (n = 3, **P < 0.01).

Methylation status and mRNA expression level of seven repressive genes in Hela cells were performed with MeDIP-qPCR assay and Real-time PCR (Figure 4) compared with drug group(5-aza-dC, methylase inhibitors), control group and blank group. Specifically, PAX1, SFRP4 and TSLC1 possessed higher levels of methylation, while CHFR and FHIT were relatively lower. Except for FHIT and PTEN, the rest five suppressor genes CCNA1, CHFR, PAX1, SFRP4 and TSLC1 in transfection group displayed lower level of methylation status compared with control group (*P <*0.01), which decreased to 34.42%, 15.57%, 22.36%, 52.09% and 35.53%, respectively. The effects of DNMT1-siRNA and 5-aza-dC treatment were performed the identical phenomenon. The relative mRNA levels of seven repressive genes were detected by Real-time PCR. It's clear that the expression of PTEN was higher than other genes. Except for FHIT and PTEN, the expression levels of CCNA1, CHFR, PAX1, SFRP4 and TSLC1 in transfection group were higher than those in control group, with relative mRNA levels increased 6.13, 10.39, 4.98, 4.87 and 3.51 folds, respectively.

### Effects of DNMT1 silencing on gene demethylation and mRNA expression level in Siha cell

**Figure 5:**
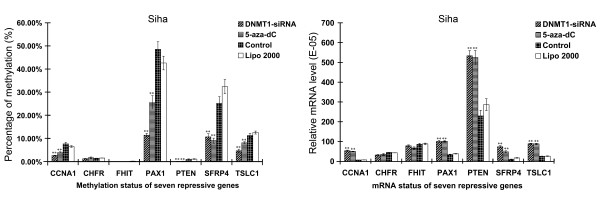
**Effects of DNMT1 silencing on gene methylation and mRNA expression of seven tumor suppressor genes in Siha cells assayed by MeDIP combined with Real-Time PCR**. Except for FHIT and CHFR, the rest five suppressor genes CCNA1, PTEN, PAX1, SFRP4 and TSLC1 in transfected group displayed lower level of methylation with increased mRNA expression when compared with control group. (n = 3, **P < 0.01).

Figure 5 showed the methylation status and mRNA levels in Siha cells were similar to those in Hell cells. PAX1, SFRP4 and TSLC1 possessed higher level of methylation status, while PTEN and FHIT were relatively lower. Except for FHIT and CHFR, the rest five repressor genes CCNA1, PAX1, PTEN, SFRP4 and TSLC1 in transfection group displayed lower level of methylation compared with control group (*P <*0.01), which decreased to 35.21%, 23.75%, 19.51%, 33.15% and 38.04%, respectively. Furthermore, the relative mRNA expression level of PTEN was higher than other genes. Except for FHIT and CHFR, the mRNA expression levels of CCNA1, PAX1, PTEN, SFRP4 and TSLC1 in transfection group were higher than those in control group, with relative mRNA levels increased 7.22, 2.88, 2.32, 7.04 and 3.47 folds, respectively.

## Discussion

DNMT1 silencing in cervical cancer cells could induce re-expression of most tumor suppressor genes by demethylating its promoter region, and co-silencing of DNMT1 and DNMT3b might perform a greater inhibitory effect on tumorigenesis [3]. Sowinska [4] demonstrated that combined DNMT1 and DNMT3b siRNAs could enhance promoter demethylation and re-expression of CXCL12 in MCF-7 breast cancer as well as AsPC1 in pancreatic carcinoma cell lines, and suggested that they acted synergistically in inhibiting CpG island hypermethylation of tumor suppressor genes. Rhee et al [5] reported that DNMT3b deletion in a colorectal cancer cell line reduced global DNA methylation by less than 3%, but co-silencing of both DNMT1 and DNMT3b nearly eliminated methyltransferase activity, and reduced genomic DNA methylation by greater than 95%. Thus, DNMT1 and DNMT3b play the significant role in promoter methylation of tumor suppressor genes and tumorigenesis in its early status. Currently, functions and mechanisms of DNMTs in cervical cancer cells remained unclear, and whether DNMT1 and DNMT3b act synergistically or through other ways exploration efforts were still required study.

In human bladder cancer cells, selective depletion of DNMT1 with siRNA induced demethylation and reactivation of the silenced tumor-suppressor gene CDKN2A [6]. RNAi-mediated knockdown of DNMT1 resulted in significant reduction of promoter methylation and re-expression of RASSF1A, p16, and HPP1 in HCC1954 breast cancer cells [7]. In ovarian cancer cell line CP70, DNMT1 siRNA treatment led to a partial removal of DNA methylation from three inactive promoter CpG islands, TWIST, RASSF1A, and HIN-1, and restored the expression of these genes [8]. Thus, RNAi-mediated DNMT1 depletion in different tumor cells could induce demethylation of various tumor suppressor genes and enhance re-expression. However, contradictory results were reported even in the same cell line. Ting et al [9] found that hypermethylation of CDKN2A, SFPR1, GATA4 and GATA5 were still maintained in HCT116 colorectal cancer cells after transiently or stably depleted of DNMT1, and suggested that DNMT1 might not play the dominant effect which caused hypermethylation of CpG islands in tumor suppressor genes. Knockout of DNMT1 in HCT116 cells by homologous recombination only reduced global DNA methylation by 20% and p16 maintained completely methylated status. Besides, methylations of HMLH1, p16 and CDH1 in gastric-cancer tissue samples at different progress periods do not correlate with the expression of DNMT1 directly [10]. Therefore, whether over-expression of DNMT1 accounts for the only or key causes of hypermethylation of tumor suppressor genes remains to be confirmed.

Currently, correlation between methlylation and mRNA expression still remains unclear. In our study, methylation status of five suppressor genes (such as PAX1) in transfection group was significantly lower than that in control group or blank control, and the mRNA expression levels were higher as compared to the two types of control, suggesting that lower level of methylation facilitates mRNA expression. This trend was confirmed when CCNA1, SFRP4, TSLC1 and CHFR in Hela cells and CCNA1, PTEN, SFRP4 and TSLC1 in Siha cells were analyzed.

Surprisingly, transfection did not affect the methylation status and mRNA expression of FHIT and PTEN in Hela cells and FHIT and CHFR in Siha cells in our study, even though both of these two genes might achieve high mRNA expression through low methylation. It was previously reported that there was no PTEN mutation in 63 cases of squamous cervical carcinomas, but 58% of the cases showed high methylation of PTEN promoter [11,12]. Wu et al [13] reported that FHIT was highly methylated in Hela, C33A and Siha cervical cancer cells, and that aberrant methylation of the FHIT gene might be a key mechanism for cervical tumorigenesis, which could be reactivated and whose tumor suppressing function could be restored by treatment of demethylating agent. Banno et al [14] reported that cervical smears showed aberrant methylation of CHFR in 12.3% of adenocarcinoma specimens, while aberrant DNA methylation was not detected in normal cervical cells. These researches demonstrated us that FHIT and PTEN in Hela cells and FHIT and CHFR in Siha cells might have the other regulation pathways for carcinogenesis or transcription control, and which needs more tests of cervical cancer cells and clinical specimens.

Apart from DNMT1 silencing, we treated Hela and Siha cells with 5-aza-dC, which revealed the similar results with transfection group. Five repressor genes were demethylated to various degrees and the mRNA expressions were also increased. These results are in accordance with the findings of other reports [15-19], which could be important in the development of new and effective strategy in cervical treatment.

## Conclusions

In conclusion, our study demonstrates that DNMT1 silencing could suppress proliferation and induce apoptosis of Hela and Siha cells. DNMT1-siRNA induces demethylation of five tumor suppressor genes, including CCNA1, CHFR, PAX1, SFRP4 and TSLC1 in Hela cells and CCNA1, PTEN, PAX1, SFRP4 and TSLC1 in Siha cells, and enhances their mRNA expression. In a word, DNMT1 represents an important potential diagnostic and therapeutic target for cervical cancer.

## Competing interests

The authors declare that they have no competing interests.

## Authors' contributions

YZ carried out the molecular genetic studies and wrote the manuscript, FQC and RC analyzed the dates and informations. YHS gave assistance with technical performance, SYZ contributed to the writing of the manuscript, TYL designed the study and revised the manuscript. All authors read and approved the final manuscript.
